# Clinical features of COVID-19-related encephalitis: comparison with the features of herpes virus encephalitis and autoimmune encephalitis

**DOI:** 10.1007/s10072-024-07587-5

**Published:** 2024-05-22

**Authors:** Yue Cui, Zhongyun Chen, Yu Kong, Yingtao Wang, Yihao Wang, Jing Zhang, Lin Wang, Jiatang Zhang, Wei Sun, Liyong Wu

**Affiliations:** 1 Department of Neurology, Xuanwu Hospital, Capital Medical University, Beijing, China; 2 Department of Neurology, The First Medical Centre, Chinese PLA General Hospital, Beijing, China

**Keywords:** COVID-19-related encephalitis, SARS-CoV-2 infection, Intracranial inflammation

## Abstract

**Introduction:**

Identifying coronavirus disease 2019 (COVID-19)-related encephalitis without clear etiological evidence is clinically challenging. The distinctions between this condition and other prevalent encephalitis types remain unknown. Therefore, we aimed to explore the similarities and differences in the clinical characteristics of COVID-19-related encephalitis and other encephalitis types.

**Methods:**

Adult patients with encephalitis admitted to the neurology department at Xuanwu Hospital were enrolled and categorized into the following six groups based on the results of metagenomic next-generation sequencing and autoimmune antibody detection in cerebrospinal fluid (CSF): COVID-19-related encephalitis (*n* = 36), herpes simplex virus type 1 encephalitis (HSV-1 encephalitis; *n* = 28), human herpesvirus 3 encephalitis (HHV-3 encephalitis; *n* = 10), NMDAR-antibody encephalitis (*n* = 18), LGI1-antibody encephalitis (*n* = 12), and GABAB-antibody encephalitis (*n* = 8).

**Results:**

The predominant characteristics of COVID-19-related encephalitis include a low incidence of seizures (38.9%), cognitive defects (30.6%), and meningeal irritation signs (8.3%). Compared with HSV-1 and HHV-3 encephalitis, COVID-19-related encephalitis exhibited lower white blood cell count (2.5 count/mm^3^), protein (32.2 mg/dL), and immunoglobulin M, G, and A levels (0.09, 3.2, and 0.46 mg/dL, respectively) in the CSF tests. Abnormal imaging findings were present in only 36.1% of COVID-19-related encephalitis cases, mostly showing diffuse inflammation scattered in various parts, which differed from HSV-1 encephalitis. Additionally, COVID-19-related encephalitis exhibited significant differences in clinical symptoms and CSF white blood cell counts compared with NMDAR-antibody encephalitis; however, it showed limited differences compared with LGI1-antibody and GABAB-antibody encephalitis.

**Discussion:**

COVID-19-related encephalitis and herpes virus or autoimmune encephalitis differ clinically. Symptoms and auxiliary examinations can be used as distinguishing tools.

**Supplementary Information:**

The online version contains supplementary material available at 10.1007/s10072-024-07587-5.

## Introduction

Since the initial detection of severe acute respiratory syndrome coronavirus 2 (SARS-CoV-2) in December 2019, the virus has rapidly spread worldwide, infecting more than 670 million people with over 6 million deaths [1]. Despite limited published studies, China encountered one of the most severe outbreaks of coronavirus disease 2019 (COVID-19) from December 2022 to February 2023, resulting in several hospitalizations due to complications [1]. Under these circumstances, COVID-19-related encephalitis, a severe neurological complication of COVID-19 that remains inadequately researched, has attracted attention.

COVID-19-related encephalitis is an inflammation of the brain parenchyma, occurring simultaneously with or within a few months of SARS-CoV-2 infection, with or without respiratory infection symptoms [2, 3]. This particular encephalitis is not directly caused by virus replication in the central nervous system; it is a neurological syndrome after infection, which differs from general viral encephalitis [4, 5]. Despite its prevalence of less than 1%, it is a serious complication with high mortality and disability rates that may affect the prognosis of patients with COVID-19 [3, 6, 7]. Most available data on COVID-19-related encephalitis originated from published case reports or case series from Europe and the Americas, which has restricted our understanding of the disease [3]. Secondary or retrospective studies have suggested that COVID-19-related encephalitis exhibits symptoms, cerebrospinal fluid (CSF) white blood cell counts, and CSF protein levels similar to those in herpes virus encephalitis or autoimmune encephalitis [3, 8–10]. However, there remain insufficient studies describing the clinical features of COVID-19-related encephalitis to help facilitate its differentiation from the common herpes virus or autoimmune encephalitis.

Therefore, we aimed to describe the clinical characteristics of COVID-19-related encephalitis and its differences from other types of encephalitis. To supplement reported cases of COVID-19-related encephalitis in Asia, we included patients with encephalitis admitted to the neurology department of Xuanwu Hospital, mainly during the outbreak of SARS-CoV-2 infection. After screening, the clinical characteristics of patients with COVID-19-related encephalitis were compared with those of patients with confirmed herpes simplex virus type 1 encephalitis (HSV-1 encephalitis), human herpesvirus 3 encephalitis (HHV-3 encephalitis), NMDAR-antibody encephalitis, LGI1-antibody encephalitis, and GABA_B_-antibody encephalitis.

## Materials and methods

### Study population

Between December 7, 2022, and June 28, 2023, all patients with suspected encephalitic symptoms admitted to the Department of Neurology at Xuanwu Hospital were screened. Patients were enrolled if older than 18 years, had a clear SARS-CoV-2 infection within the last two months from the date of enrollment, and met the diagnostic criteria for encephalitis established by Venkatesan [11] in 2013. SARS-CoV-2 infection was identified using reverse-transcription polymerase chain reaction or antigen detection in throat and nasopharyngeal swabs. Fever was excluded from the minor criteria for encephalitis due to its frequent association with infection in all systems. All cases were independently diagnosed by two neurologists and confirmed by an internist and intensivist, ensuring that neurological symptoms and ancillary findings were not attributable to other systemic diseases.

All patients underwent CSF metagenomic next-generation sequencing (mNGS), CSF, and serum autoimmune antibody tests. Those with negative results in the first test were retested after 5–14 days to avoid false-negative results (unless early death occurred). After a thorough examination, patients were excluded if they met the following criteria: 1) presence of herpes virus type 4 detected by mNGS testing of CSF, as this virus is usually incidental to other infectious or immune encephalitis; 2) history of confirmed or suspected encephalitis, with a possibility of disease recurrence; 3) positive results in both mNGS and antibody tests; and 4) a definite diagnosis of other central nervous system diseases.

Based on the results of CSF mNGS and CSF and serum neuronal antibodies, patients were categorized into the following six groups: COVID-19-related encephalitis, HSV-1 encephalitis, HHV-3 encephalitis, NMDAR-antibody encephalitis, LGI1-antibody encephalitis, and GABA_B_-antibody encephalitis. The diagnosis of COVID-19-related encephalitis was based on a temporal relationship with COVID-19 and a negative search for other causative factors [5]. The COVID-19-related encephalitis group comprised patients with no detectable pathogens or autoimmune antibodies other than those against SARS-CoV-2. The HSV-1 and HHV-3 encephalitis groups met the 2013 diagnostic criteria for encephalitis [11] and were confirmed by positive CSF mNGS results. The NMDAR-antibody, LGI1-antibody, and GABA_B_-antibody encephalitis groups met the 2016 diagnostic criteria for autoimmune encephalitis [12] and were diagnosed with positive serum or CSF antibodies. To supplement the control group, 22 patients with HSV-1 encephalitis, 8 with HHV-3 encephalitis, 16 with NMDAR-antibody encephalitis, 8 with LGI1-antibody encephalitis, and 6 with GABA_B_-antibody encephalitis were enrolled from the previous encephalitis database, all of whom met the same diagnostic criteria described above but had no history of COVID-19 infection or immunosuppression.

The demographic information, clinical manifestations, laboratory findings, neuroimaging results, electroencephalography (EEG) findings, treatment modalities, and outcomes of all patients were collected. The severity of encephalitis was determined using the modified Rankin scale (mRS) [13] and Glasgow Coma Scale (GCS) [14]. All patients admitted to the neurological intensive care unit were considered critically ill if met at least one of the following criteria: mechanical ventilation, intravenous catecholamine, mRS score > 4, or GCS score < 9. Written consent was obtained from families and patients’ representatives. This study was approved by the Institutional Ethical Standards Committee of Xuanwu Hospital.

### Clinical features assessment

Neurological symptoms were categorized according to the neurological assessment guidelines and common symptoms of COVID-19-related encephalitis [9, 15]. Additionally, routine blood examinations, including blood cell counts, immunoglobulin G, M, and A, complement C3 and C4, and related serum inflammatory markers such as C-reactive protein (CRP), erythrocyte sedimentation rate (ESR), fibrinogen (Fg), D-dimer, procalcitonin (PCT), and interleukin-6 (IL-6), were performed for all patients. All patients underwent serum and CSF antineuronal antibody tests, with some undergoing antinuclear antibody profiles, anticardiolipin antibody profiles, and anti-neutrophil cytoplasmic antibody tests. Cytological and biochemical testing of CSF was conducted for all patients, along with specific and non-specific immunoglobulin, oligoclonal band, and 24-h intrathecal immunoglobulin G production rate testing in most cases. Brain computed tomography (CT), magnetic resonance imaging (MRI), and EEG were performed for all patients whenever feasible. The final analysis included only individuals who completed the assessment and had imaging results (CT or MRI) within 14 days of the onset of neurological symptoms. Treatment response was defined as an increase of at least two points in the mRS score [13].

### Statistical analyses

Categorical variables were compared using the Chi-square or Fisher’s exact tests, and continuous variables were compared using the nonparametric Mann–Whitney *U* test, depending on the normality of the data distribution determined by the Shapiro–Wilk test. All statistical analyses were performed using SPSS (version 26.0; IBM Corp., Armonk, NY, USA), and all figures were generated using GraphPad Prism 7 (GraphPad Software, La Jolla, CA, USA). Statistical significance was set at *p* < 0.05.

## Results

Between December 7, 2022, and June 28, 2023, 133 patients diagnosed with encephalitis were admitted to Xuanwu Hospital’s neurology unit. After screening, 52 patients with a history of COVID-19 were included, among whom 36 were considered to have COVID-19-related encephalitis (CSF SARS-CoV-2 negative in all cases), 6 had HSV-1 encephalitis, 2 had HHV-3 encephalitis, 2 had NMDAR-antibody encephalitis, 4 had LGI1-antibody encephalitis, and 2 had GABA_B_-antibody encephalitis. Combined with the previous database, the final numbers of encephalitis cases in each group were as follows: COVID-19-related encephalitis group (*n* = 36), HSV-1 encephalitis (*n* = 28), HHV-3 encephalitis (*n* = 10), NMDAR-antibody encephalitis (*n* = 18), LGI1-antibody encephalitis (*n* = 12), and GABA_B_-antibody encephalitis (*n* = 8). Details of the characteristics of each case of COVID-19-related encephalitis are provided in Supplementary Table 1.

### Demographic characteristics and clinical presentation

The most prevalent initial neurological symptoms of COVID-19-related encephalitis were psychiatric impairment (36.1%), impaired consciousness (22.2%), seizures (13.9%), and cognitive defects (8.3%). These symptoms remained prevalent during the disease course. Additionally, more than half of the patients had fever and general symptoms during the entire disease course. In contrast, over one-fifth of the patients had focal motor deficits, increased muscle tone, and cranial nerve signs. Other less frequent symptoms observed included unsteady gait (11.1%), dizziness (11.1%), limb tremors (11.1%), involuntary movements (8.3%), dysautonomia (2.8%), and sensory defects (2.8%).

Compared with other types of encephalitis groups, the most typical characteristics of the COVID-19-related encephalitis group were a lower incidence of seizure (HSV-1 encephalitis group: χ^2^ = 6.70, *p* = 0.01; NMDAR-antibody encephalitis group: χ^2^ = 5.80, *p* = 0.02), cognitive defects (HSV-1 encephalitis group: χ^2^ = 12.44, *p* < 0.01; NMDAR-antibody encephalitis group: χ^2^ = 8.44, *p* < 0.01; LGI1-antibody encephalitis group: χ^2^ = 5.60, *p* = 0.02) and meningeal irritation signs (HSV-1 encephalitis group: χ^2^ = 5.70, *p* = 0.02; HHV-3 encephalitis group: χ^2^ = 3.88, *p* = 0.049; GABA_B_-antibody encephalitis group: χ^2^ = 5.67, *p* = 0.02) during the entire disease course. Besides, the COVID-19-related encephalitis group was more likely to present with fever during the disease course than the LGI1-antibody encephalitis group (χ^2^ = 11.19, *p* < 0.01) and less likely to present with psychiatric impairments during the disease course compared with NMDAR-antibody encephalitis group (χ^2^ = 6.20, *p* = 0.01). Detailed demographic characteristics and clinical presentations of each encephalitis group are listed in Table 1.

**Table 1:** Demographic characteristics and clinical presentation according to each encephalitis group

	COVID-19 related encephalitis (*n* = 36)	HSV-1 encephalitis (*n* = 28)	HHV-3 encephalitis (*n* = 10)	NMDAR-antibody encephalitis (*n* = 18)	LGI1-antibody encephalitis (*n* = 12)	GABA_B_-antibody encephalitis (*n* = 8)
Age of onset (year)	48.5 (38.0–62.5)	50.5 (44.0–62.3)	56.5 (38.3–67.8)	31.0 (25.8–36.5)*	64.0 (46.3–72.5)*	59.0 (57.5–62.5)
Male Sex	22 (61.1%)	19 (67.9%)	7 (70.0%)	8 (44.4%)	5 (41.7%)	7 (87.5%)
Neurological symptoms at first						
Psychiatric impairments	13 (36.1%)	12 (42.9%)	1 (10.0%)	8 (44.4%)	2 (16.7%)	1 (12.5%)
Impaired consciousness	8 (22.2%)	4 (14.3%)	2 (20.0%)	0 (0.0%)	1 (8.3%)	0 (0.0%)
Seizure	5 (13.9%)	5 (17.9%)	0 (0.0%)	6 (33.3%)	7 (58.3%)*	4 (50.0%)
Cognitive defects	3 (8.3%)	5 (17.9%)	2 (20.0%)	4 (22.2%)	2 (16.7%)	2 (25.0%)
Symptoms during the disease course						
Fever	25 (69.4%)	23 (82.1%)	6 (60.0%)	11 (61.1%)	1 (8.3%)*	2 (25.0%)
Psychiatric impairments	18 (50.0%)	18 (64.3%)	2 (20.0%)	16 (88.9%)*	5 (41.7%)	5 (62.5%)
Seizure	14 (38.9%)	20 (71.4%)*	1 (10.0%)	14 (77.8%)*	9 (75.0%)	5 (62.5%)
Cognitive defects	11 (30.6%)	21 (75.0%)*	4 (40.0%)	13 (72.2%)*	9 (75.0%)*	6 (75.0%)
Focal motor deficit/increased muscle tone	9 (25.0%)	5 (17.9%)	3 (30.0%)	1 (5.6%)	3 (25.0%)	1 (12.5%)
Cranial nerve signs	8 (22.2%)	3 (10.7%)	5 (50.0%)	3 (16.7%)	1 (8.3%)	1 (12.5%)
Meningeal irritation signs	3 (8.3%)	10 (35.7%)*	4 (40.0%)*	6 (33.3%)	1 (8.3%)	4 (50.0%)*
Critically illness	11 (30.6%)	12 (42.9%)	3 (30.0%)	7 (38.9%)	1 (8.3%)	5 (62.5%)
Status epilepticus	9 (25.0%)	7 (25.0%)	2 (20.0%)	5 (27.8%)	0 (0.0%)	4 (50.0%)
Invasive mechanical ventilation	7 (19.4%)	5 (17.9%)	2 (20.0%)	4 (22.2%)	0 (0.0%)	3 (37.5%)
Outcome						
Highest mRS during the disease	3.5 (3.0–5.0)	3.5 (3.0–5.0)	3.5 (3.0–4.3)	4.0 (3.0–5.0)	3.0 (3.0–3.8)	5.0 (4.0–5.0)
Median mRS at discharge	2.0 (1.0–2.0)	2.0 (1.0–3.8)	2.0 (1.0–4.0)	3.0 (2.0–5.0)*	1.0 (1.0–2.8)	4.0 (4.0–5.0)*
Treatment response	30 (83.3%)	23 (82.1%)	8 (80.0%)	16 (88.9%)	12 (100.0%)	2 (25.0%)*

Data are presented as No.(%) or median (quartile). *Abbreviations*: *COVID-19* coronavirus disease 2019; Comparisons were made between the COVID-19 related encephalitis group and HSV-1 encephalitis group, HHV-3 encephalitis group, NMDAR-antibody encephalitis group, LGI1-antibody encephalitis group and GABA_B_-antibody encephalitis group respectively. ^*^There were statistical differences between the COVID-19 related encephalitis group and the control encephalitis group

### Comorbidities and complications

Of all patients with COVID-19-related encephalitis, 25 (69.4%) had chronic comorbidities, of whom 8 (22.2%) had only one chronic comorbidity, 12 (33.3%) had two or three, and 5 (13.9%) had more than three. Hypertension was the most common chronic comorbidity, affecting 33.3% of the patients; moreover, 26 patients with COVID-19-related encephalitis (72.2%) experienced complications, of whom 5 (13.9%) had only one complication, 11 (30.6%) had two or three, and 10 (27.8%) had more than three. The most common complications were bacterial and fungal respiratory infections (52.8%). Due to the small sample size of each group, no further group comparisons were performed.

### Blood and cerebrospinal fluid examinations

The white blood cell count in the COVID-19-related encephalitis group was 5.7 (4.4–8.0) count/μL, with only six patients falling outside the normal range. All hematological inflammatory markers, including CRP (57.1%), ESR (40.7%), fibrinogen (54.5%), D-dimer (68.8%), PCT (66.7%), and IL-6 (60.7%), were elevated in approximately half of the patients in the COVID-19-related encephalitis group. The interval between the first onset of neurological symptoms and CSF examination in patients with COVID-19-related encephalitis was 8.0 (4.25–12.75) days. Additionally, 44.1% of patients with COVID-19-related encephalitis had abnormally increased intracranial pressure, while 50.0% had pleocytosis or high CSF protein levels.

Compared with HSV-1 and HHV-3 encephalitis groups, the CSF examination results of the COVID-19-related encephalitis group were significantly different, characterized by lower white blood cell count (2.5, 1.0–8.8 count/mm^3^), protein (32.2, 24.5–52.5 mg/dL) and immunoglobulin M, G, and A levels (0.09, 0.05–0.13 mg/dL; 3.2, 2.4–6.2 mg/dL; 0.46, 0.21–0.66 mg/dL). However, compared with the autoimmune encephalitis group, especially the LGI1-antibody encephalitis group and GABA_B_-antibody encephalitis group, limited differences were observed in the results of blood and CSF examinations in the COVID-19-related encephalitis group. Detailed results of the blood and CSF examinations for each encephalitis group are shown in Table 2.

**Table 2:** The main results of auxiliary examinations according to each encephalitis group

	COVID-19 related encephalitis (*n* = 36)	HSV-1 encephalitis (*n* = 28)	HHV-3 encephalitis (*n* = 10)	NMDAR-antibody encephalitis (*n* = 18)	LGI1-antibody encephalitis (*n* = 12)	GABA_B_-antibody encephalitis (*n* = 8)
Blood examination						
White blood cell (count/μL)	5.7 (4.4–8.0)	7.6 (6.1–9.9) *	6.3 (4.9–10.4)	9.0 (6.8–11.2) *	8.2 (6.1–10.2) *	12.5 (9.6–13.4) *
Granulocyte (%)	65.7 (55.0–78.7)	78.0 (68.6–84.5)*	66.2 (57.2–81.0)	73.7 (67.9–81.4) *	66.6 (62.5–78.2)	86.2 (80.9–87.3) *
Lymphocyte (%)	22.8 (12.7–32.8)	15.7 (8.5–20.9) *	23.7 (11.2–34.5)	18.5 (14.3–22.6)	25.5 (15.0–27.6)	8.8 (5.3–11.4) *
CRP (mg/L)	19.0 (2.0–49.0)	8.2 (3.0–88.6)	26.0 (2.0–46.5)	6.0 (2.3–29.3)	4.0 (2.0–9.9)	72.5 (4.8–153.0)
ESR (mm/h)	10.0 (5.0–39.0)	9.0 (8.0–16.0)	10.0 (3.0–28.0)	10.0 (5.0–27.5)	10.5 (6.0–23.0)	26.0 (12.5–47.0)
Fibrinogen (g/L)	4.1 (3.1–6.0)	4.1 (3.4–5.3)	3.3 (3.0–5.3)	3.1 (2.4–4.1) *	3.8 (3.0–4.7)	4.4 (2.9–5.4)
D-dimer (ug/mL)	1.4 (0.4–3.7)	1.7 (0.9–5.7)	1.8 (0.2–7.2)	0.5 (0.3–1.2) *	0.3 (0.2–0.9) *	2.5 (0.5–2.9)
PCT (ng/mL)	0.08 (0.04–0.17)	0.16 (0.05–2.04)	0.08 (0.03–0.35)	0.04 (0.03–0.07) *	0.04 (0.02–0.26)	0.06 (0.04–0.57)
IL-6 (pg/mL)	14.5 (2.6–30.3)	54.3 (18.4–106.1) *	4.0 (2.4–52.8)	10.9 (4.1–37.2)	5.1 (4.2–12.8)	8.5 (5.4–28.0)
IgM (mg/dL)	0.9 (0.6–1.2)	0.9 (0.6–1.5)	0.9 (0.8–1.5)	1.0 (0.9–1.3)	1.0 (0.5–1.3)	0.7 (0.5–1.5)
IgG (mg/dL)	10.8 (9.5–13.9)	10.4 (7.6–11.7)	8.5 (7.3–9.4) *	10.5 (8.9–16.8)	12.3 (9.6–13.7)	9.0 (7.9–15.6)
IgA (mg/dL)	2.5 (1.9–3.2)	2.2 (1.6–3.1)	1.9 (1.4–2.4)	2.0 (1.4–2.6) *	2.6 (1.9–3.8)	2.3 (1.3–2.4)
Complement C3 (g/L)	0.95 (0.82–1.10)	0.84 (0.80–0.93)	0.86 (0.71–0.97)	0.86 (0.79–0.96)	0.85 (0.69–0.96)	0.95 (0.86–1.27)
Complement C4 (g/L)	0.22 (0.19–0.28)	0.24 (0.20–0.26)	0.25 (0.22–0.32)	0.21 (0.17–0.25)	0.21 (0.17–0.27)	0.30 (0.17–0.37)
Cerebrospinal fluid examination						
The interval between CSF examination and the onset of neurological symptoms (days)	8.0 (4.25–12.75)	8.0 (6.0–11.0)	11.0 (6.0–19.0)	14.5 (10.0–18.0) *	18.5 (14.3–26.3) *	16.0 (11.8–22.0) *
Intracranial pressure (mmHg)	170.0 (113.8–217.5)	200.0 (182.3–230.0) *	150.0 (105.0–215.0)	150.0 (110.0–183.5)	132.5 (115.0–147.5)	160.0 (120.0–210.0)
Leukocyte (count/mm3)	2.5 (1.0–8.8)	59.0 (18.0–109.3) *	109.0 (50.5–505.3) *	30.0 (10.3–186.5) *	2.0 (1.0–6.5)	19.0 (4.5–24.8)
Pleocytosis (number)	12/36 (33.3%)	26/28 (92.9%)*	10/10 (100.0%)*	16/18 (88.9%)*	3/12 (25.0%)	6/8 (75.0%)
Monocytes (%)	99.0 (75.0–100.0)	98.0 (93.9–99.0)	96.5 (89.3–98.2)	95.1 (80.2–98.3)	100.0 (98.5–100.0) *	98.0 (82.9–99.7)
Protein (mg/dL)	32.2 (24.5–52.5)	62.0 (45.0–119.0) *	85.6 (54.7–123.9) *	41.9 (22.8–55.2)	36.3 (23.4–44.2)	39.5 (28.0–47.0)
High protein (number)	11/36 (30.6%)	20/28 (71.4%)*	8/10 (80.0%)*	7/18 (38.9%)	2/12 (16.7%)	3/8 (37.5%)
Pleocytosis or high protein	18/36 (50.0%)	26/28 (92.9%)*	10/10 (100.0%)*	16/18 (88.9%)*	4/12 (33.3%)	7/8 (87.5%)
Glucose (mg/dL)	63.2 (57.0–81.8)	57.2 (49.9–80.7)	54.3 (42.2–63.1) *	65.9 (59.6–72.5)	78.1 (63.7–97.3)	80.6 (52.7–116.1)
Chlorin (mmol/L)	127.3 (125.0–130.0)	123.0 (120.0–126.3) *	118.9 (111.2–123.8) *	125.4 (119.0–129.3)	124.0 (115.0–129.5)	122.0 (120.0–123.8) *
IgM (mg/dL)	0.09 (0.05–0.13)	0.33 (0.17–1.21) *	0.57 (0.27–1.84) *	0.11 (0.03–0.82)	0.05 (0.03–0.15)	0.24 (0.11–0.31) *
IgG (mg/dL)	3.2 (2.4–6.2)	9.3 (4.8–15.7) *	12.4 (5.9–19.2) *	3.9 (2.3–11.1)	4.6 (2.1–6.0)	7.8 (4.9–10.4) *
IgA (mg/dL)	0.46 (0.21–0.66)	1.09 (0.65–2.84) *	1.73 (1.09–2.16) *	0.60 (0.27–0.95)	0.54 (0.35–1.30)	0.65 (0.36–0.92)
Intrathecal IgG synthesis (mg/24 h)	1.7 (0.0–4.0)	15.1 (2.9–51.4) *	13.0 (7.3–69.5)	2.9 (1.3–28.0)	4.6 (1.4–7.4) *	9.1 (1.9–22.5) *
Imaging examination and electroencephalogram						
MRI positivity (number)	11/34 (32.4%)	22/24 (91.7%)*	5/9 (55.6%)	8/16 (50.0%)	7/12 (58.3%)	4/8 (50.0%)
Image positivity (number)	13/36 (36.1%)	22/28 (78.6%)*	5/10 (50.0%)	8/18 (44.4%)	7/12 (58.3%)	4/8 (50.0%)
Frontal lobe	4/36 (11.1%)	7/28 (25.0%)	2/10 (20.0%)	6/18 (33.3%)	0/12 (0.0%)	2/8 (25.0%)
Parietal lobe	4/36 (11.1%)	3/28 (10.7%)	2/10 (20.0%)	3/18 (16.7%)	0/12 (0.0%)	1/8 (12.5%)
Temporal lobe	4/36 (11.1%)	19/28 (67.9%)*	2/10 (20.0%)	6/18 (33.3%)	3/12 (25.0%)	2/8 (25.0%)
Occipital lobe	1/36 (2.8%)	1/28 (3.6%)	1/10 (10.0%)	3/18 (16.7%)	2/12 (16.7%)	0/8 (0.0%)
Insular lobe	2/36 (5.6%)	15/28 (53.6%)*	0/10 (0.0%)	3/18 (16.7%)	0/12 (0.0%)	2/8 (25.0%)
Limbic lobe	3/36 (8.3%)	13/28 (46.4%)*	1/10 (10.0%)	1/18 (5.6%)	4/12 (33.3%)	0/8 (0.0%)
Brainstem	4/36 (11.1%)	0/28 (0.0%)	2/10 (20.0%)	1/18 (5.6%)	0/12 (0.0%)	0/8 (0.0%)
Basal ganglia	3/36 (8.3%)	0/28 (0.0%)	2/10 (20.0%)	0/18 (0.0%)	2/12 (16.7%)	0/8 (0.0%)
Thalamus	1/36 (2.8%)	5/28 (17.9%)	1/10 (10.0%)	0/18 (0.0%)	0/12 (0.0%)	0/8 (0.0%)
Deep white matter	3/36 (8.3%)	4/28 (14.3%)	1/10 (10.0%)	2/18 (11.1%)	0/12 (0.0%)	0/8 (0.0%)
Cerebellum	1/36 (2.8%)	0/28 (0.0%)	0/10 (0.0%)	1/18 (5.6%)	0/12 (0.0%)	1/8 (12.5%)
Spinal cord	0/36 (0.0%)	0/28 (0.0%)	1/10 (10.0%)	0/18 (0.0%)	0/12 (0.0%)	0/8 (0.0%)
EEG positivity (number)	10/16 (62.5%)	16/16 (100.0%)*	1/10 (10.0%)*	7/7 (100.0%)	4/5 (80.0%)	5/5 (100.0%)

Data are presented as No./Total No. (%) or median (quartile). *Abbreviations*: *COVID-19* coronavirus disease 2019, *CRP* C reactive protein, *ESR* erythrocyte sedimentation rate, *PCT* procalcitonin, *IL-6* interleukin-6, *Ig* immune globulin, *CSF* Cerebrospinal fluid, *MRI* magnetic resonance imaging, *EEG* electroencephalogram. Image positivity included CT positivity and MRI positivity. Comparisons were made between the COVID-19 related encephalitis group and HSV-1 encephalitis group, HHV-3 encephalitis group, NMDAR-antibody encephalitis group, LGI1-antibody encephalitis group and GABA_B_-antibody encephalitis group respectively. *There were statistical differences between the COVID-19 related encephalitis group and the control encephalitis group

### Imaging examination and electroencephalography

**Fig. 1:**
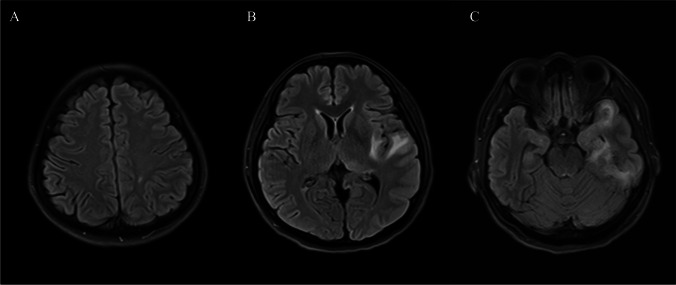
Typical imaging findings of COVID-19-related encephalitis. **A**–**C**: multiple abnormal signals were found in the frontal lobe, parietal lobe, temporal lobe, insula lobe, hippocampus, and thalamus on the head MRI T2 FLAIR phase

Among the 36 patients with COVID-19-related encephalitis, 36.1% showed inflammatory imaging abnormalities, with two patients undergoing CT examination and the others undergoing MRI examination. The proportion of MRI and image positivity in the COVID-19-related encephalitis group was significantly lower (χ^2^ = 17.84, *p* < 0.01; χ^2^ = 11.46, *p* < 0.01), and the distribution of inflammation was more scattered than those of HSV-1 encephalitis group. The typical imaging findings of COVID-19-related encephalitis are shown in Fig. 1.

EEG examination was performed in 16 patients with COVID-19-related encephalitis, of whom 62.5% showed non-specific abnormalities, which was significantly higher than that in the HSV-1 encephalitis group but significantly lower than that in the HHV-3 encephalitis group. Detailed results of the imaging examination and EEG for each encephalitis group are shown in Table 2.

### Comparison of COVID-19-related encephalitis with non-critical illness and critical illness

Compared with non-critically ill patients with COVID-19-related encephalitis, critically ill patients exhibited significantly increased white blood cell count and percentage of neutrophils (U = 219.0, *p* < 0.01; U = 241.0, *p* < 0.01) and a significantly decreased percentage of lymphocytes (U = 13.0, *p* < 0.01). Additionally, most hematological inflammatory indicators in critically ill patients with COVID-19-related encephalitis were significantly higher than those in non-critically ill patients. However, in the CSF tests, only intracranial pressure and glucose levels differed significantly between the two groups (U = 184.0, *p* = 0.03; U = 233.0, *p* < 0.01). No significant differences were observed in the positivity rates of imaging examinations between the two groups. Detailed results are presented in Table 3.

**Table 3:** Comparison of COVID-19 related encephalitis with non-critical illness and critical illness

	Non-critical illness (*n* = 25)	Critical illness (*n* = 11)	U/χ^2^	p
Blood examination				
White blood cell (count/μL)	5.1 (4.3–6.6)	8.0 (5.7–13.8)	219.0	< 0.01^*^
Granulocyte (%)	60.8 (49.7–66.7)	80.5 (67.5–91.3)	241.0	< 0.01^*^
Lymphocyte (%)	29.5 (22.1–35.1)	8.2 (5.6–17.2)	13.0	< 0.01^*^
CRP (mg/L)	4.5 (1.0–19.0)	81.0 (33.0–89.0)	247.0	< 0.01^*^
ESR (mm/h)	10.0 (3.5–36.5)	16.0 (9.0–30.0)	37.5	0.25
Fibrinogen (g/L)	3.6 (2.8–4.5)	6.2 (5.5–7.3)	215.5	< 0.01^*^
D-dimer (ug/mL)	0.6 (0.2–1.8)	3.8 (2.3–7.8)	204.0	< 0.01^*^
PCT (ng/mL)	0.05 (0.04–0.08)	0.17 (0.10–0.62)	161.5	< 0.01^*^
IL-6 (pg/mL)	3.3 (1.8–16.4)	30.3 (24.6–53.1)	162.0	< 0.01^*^
IgM (mg/dL)	0.9 (0.6–1.2)	0.9 (0.6–1.2)	126.5	0.71
IgG (mg/dL)	10.5 (9.5–13.5)	11.6 (9.4–19.7)	164.0	0.36
IgA (mg/dL)	2.5 (2.1–3.4)	2.5 (1.5–3.0)	102.5	0.23
Complement C3 (g/L)	0.89 (0.80–1.00)	1.16 (0.84–1.28)	209.0	0.02^*^
Complement C4 (g/L)	0.21 (0.17–0.27)	0.27 (0.22–0.29)	208.5	0.02^*^
Cerebrospinal fluid examination				
Intracranial pressure (mmHg)	160.0 (110.0–190.0)	210.0 (150.0–280.0)	184.0	0.03^*^
Leukocyte (count/mm3)	2.0 (1.0–8.0)	3.0 (1.0–13.0)	149.5	0.67
Pleocytosis (number)	8/25 (32.0%)	4/11 (36.4%)	**-**	1.00
Monocytes (%)	100.0 (75.0–100.0)	82.9 (50.0–100.0)	71.5	0.14
Protein (mg/dL)	37.0 (21.0–53.2)	27.8 (25.3–39.7)	124.0	0.64
High protein (number)	9/25 (36.0%)	2/11 (18.2%)	-	0.44
Pleocytosis or high protein	12/25 (48.0%)	6/11 (54.5%)	-	1.00
Glucose (mg/dL)	61.0 (55.9–70.1)	102.2 (68.9–140.4)	233.0	< 0.01^*^
Chlorin (mmol/L)	127 (124.0–129.5)	129.0 (126.0–136.0)	185.0	0.10
IgM (mg/dL)	0.09 (0.03–0.13)	0.11 (0.07–0.21)	115.0	0.35
IgG (mg/dL)	2.9 (2.2–5.4)	4.2 (2.7–10.7)	121.0	0.23
IgA (mg/dL)	0.47 (0.19–0.67)	0.46 (0.20–0.81)	104.5	0.65
Intrathecal IgG synthesis (mg/24 h)	1.4 (0.0–4.2)	2.4 (0.0–4.1)	78.0	0.88
Imaging examination				
Image positivity (number)	6/25 (24.0%)	7/11 (63.6%)	-	0.06

Data are presented as No./Total No. (%) or median (quartile). *Abbreviations*: *CRP* C reactive protein, *ESR* erythrocyte sedimentation rate, *PCT* procalcitonin, *IL-6* interleukin-6, *Ig* immune globulin, *CSF* Cerebrospinal fluid. Image positivity included CT positivity and MRI positivity. ^*^represents a statistically significant difference

## Discussion

Several systematic reviews and meta-analyses have reported only approximately 100 cases of COVID-19-related encephalitis, with a notably small number of Asian patients [16]. The 36 cases of COVID-19-related encephalitis reported in this study address a gap in case reports from Asia. To our knowledge, this is the first study to explore the similarities and differences in the clinical characteristics of COVID-19-related encephalitis and other types of encephalitis.

Currently, the management of SARS-CoV-2 infection has entered the normalization stage in most parts of the world, potentially leading to a lack of pathologically confirmed history of SARS-CoV-2 infection before onset among patients with COVID-19-related encephalitis [17]. Moreover, previous studies have reported that 24% of patients with COVID-19-related encephalitis have no respiratory symptoms during the entire course of the disease, and only less than 6% of patients have detectable SARS-CoV-2 RNA in CSF [3, 18]. These conditions make it difficult for neurologists to recognize COVID-19-related encephalitis [19, 20]. However, as patients with COVID-19-related encephalitis require corticosteroids and immunological therapy that differs from that used for other types of viral encephalitis, clinically identifying possible COVID-19-related encephalitis for further testing is critical. Many systematic reviews have described the clinical characteristics of COVID-19-encephalitis, with most suggesting that its symptoms closely resemble those of herpes virus encephalitis and autoimmune encephalitis [3, 8, 9, 21]. However, owing to the lack of statistical comparisons, these descriptive clinical characteristics of COVID-19-related encephalitis do not help to distinguish its clinical differences from other types of encephalitis.

In this study, we compared the clinical features of COVID-19-related encephalitis with those of HSV-1, HHV-3, anti-NMDAR antibody, anti-LGI1 antibody, and anti-GABA_B_ antibody encephalitis, which are currently the most prevalent types of encephalitis worldwide [22]. Similar to those with postinfectious encephalitis, the patients with COVID-19-related encephalitis in this study showed a general increase in serum inflammatory factors and good response to corticosteroid therapy [4]. Importantly, similar to those with postinfectious encephalitis, the patients with COVID-19-related encephalitis showed CSF characteristics that differed from those in general viral encephalitis [4]. Specifically, the white blood cell count, protein, and immunoglobulin M, G, and A levels in the CSF of the COVID-19-related encephalitis group were significantly lower, whereas the CSF chloride level was relatively higher. Moreover, the symptoms and imaging findings of COVID-19-related and HSV-1 encephalitis differ. Epilepsy, cognitive defects, and meningeal irritation were relatively rare in patients with COVID-19-related encephalitis. Furthermore, abnormal inflammatory manifestations in the imaging of COVID-19-related encephalitis were mainly scattered in various brain regions rather than concentrated in the temporal, insular, and limbic lobes, as in HSV-1 encephalitis. These results not only provide a direction for the clinical differentiation of these diseases but also suggest that the mechanism of COVID-19-related encephalitis is different from that of herpes virus encephalitis, which is typically caused by the direct invasion of the virus and the specific immune response in the brain [23].

The differences between COVID-19-related and NMDAR-antibody encephalitis are also significant, mainly characterized by fewer psychiatric impairments, seizures, and cognitive defects during the disease course. Although the differences in blood tests between the two diseases have no clear explicable clinical value, the leukocyte count and the level of pleocytosis in the CSF of COVID-19-related encephalitis are significantly lower, reflecting their different degrees of inflammatory response. Furthermore, although slight differences exist in symptoms and blood or CSF examination between COVID-19-related encephalitis and both LGI1-antibody and GABA_B_-antibody encephalitis, it remains challenging to distinguish them based on clinical features. Imaging findings are not useful to differentiate COVID-19-related encephalitis from other types of autoimmune encephalitis.

We explored the differences in laboratory and imaging findings between non-critically ill and critically ill patients with COVID-19-related encephalitis and found that the severity of COVID-19-related encephalitis almost did not affect the inflammatory and immunological indicators of the CSF; critically ill patients only showed increased intracranial pressure and CSF glucose levels. Given the absence of contemporaneous serum glucose data, higher intracranial pressure may be the only credible CSF indicator affected by disease severity. Additionally, we found no significant difference in the imaging positivity rate between non-critically ill and critically ill patients with COVID-19-related encephalitis. However, most blood inflammation-related indicators were significantly elevated in critically ill patients. This finding suggests that the severe inflammatory response in critically ill patients with COVID-19-related encephalitis may originate from systems other than the nervous system. Previous studies have found similar elevations in blood inflammatory markers in critically ill patients with COVID-19 without neurological symptoms, possibly due to the systemic inflammatory response to COVID-19 infection [24–26].

The most significant limitation of this retrospective observational cohort study was the clinical information at the beginning of SARS-CoV-2 infection, such as the severity of respiratory symptoms and the time interval between neurological symptoms and infection, could not be extracted from the medical records. Furthermore, owing to the relatively low incidence of COVID-19-related encephalitis, the sample size of this study was small, resulting in limited statistical power. In future studies, we intend to enroll more patients with COVID-19-related encephalitis, observe their long-term prognoses, and further explore the differences in outcomes between COVID-19-related encephalitis and other types of encephalitis.

## Conclusions

COVID-19-related encephalitis is secondary to infection with highly contagious SARS-CoV-2 and should be subjected to rigorous and targeted surveillance, treatment, and isolation measures compared with other forms of encephalitis. Compared with herpes virus encephalitis and autoimmune encephalitis, COVID-19-related encephalitis exhibits distinct characteristics in terms of symptoms and clinical tests, especially CSF tests, which are helpful for clinical identification and further examination.

### Supplementary Information

Below is the link to the electronic supplementary material.

Supplementary file1 (DOCX 44 kb)

## Data Availability

The data that support the findings of this study are available on request from the corresponding author, Liyong Wu, upon reasonable request.
