# Supplementary material for: Clinical features of COVID-19-related encephalitis: comparison with the features of herpes virus encephalitis and autoimmune encephalitis
Source: Neurol Sci. 2024 May 22;45(8):3573–82. doi: 10.1007/s10072-024-07587-5 (PMC11254958; doi:10.1007/s10072-024-07587-5)
Supplement: Supplementary file 1 — Supplementary file1 (DOCX 44 kb) [file 10072_2024_7587_MOESM1_ESM.docx]

Supplementary Material

Supplementary Table 1. The characteristics of coronavirus disease 2019 related encephalitis cases

| Case | Age | Sex | Onset  (days) | Main clinical features | CSF cells  (count/mm^3^) | CSF protein  (mg/dL) | Imaging finding | EEG |
| --- | --- | --- | --- | --- | --- | --- | --- | --- |
| 1 | 63 | Female | 0 | AMS with involuntary movement | 4 | 22.2 | Bilateral temporal lobes | NA |
| 2 | 76 | Male | 10 | AMS with focal motor deficit | 6* | 53.2* | Negative | Positive |
| 3 | 47 | Male | 7 | AMS with involuntary movement | 22* | 39.7 | Negative | NA |
| 4 | 18 | Male | 0 | AMS with seizure and diplopia | 4 | 38.0 | Negative | Negative |
| 5 | 39 | Male | 2 | AMS with aphasia and focal motor deficit | 2 | 26.0 | Unilateral parietal lobe (CT) | NA |
| 6 | 58 | Female | 0 | AMS with seizure and focal motor deficit | 1 | 27.8 | Negative | NA |
| 7 | 50 | Male | <2w | AMS with seizure, dysarthria, dysphagia, and autonomic dysfunction | 2 | 30.9 | Negative | NA |
| 8 | 78 | Male | <2w | AMS with ataxia | 0 | 90.3* | Bilateral pons and cerebellum | NA |
| 9 | 69 | Female | 2 | AMS with aphasia | 1 | 28.2 | Negative | Positive |
| 10 | 41 | Female | -5 | AMS with seizure and autonomic dysfunction | 2 | 25.6 | Negative | NA |
| 11 | 64 | Male | 37 | AMS | 3 | 39.7 | Multiple regions | Positive |
| 12 | 60 | Female | -3 | AMS with dysarthria | 19* | 25.3 | Negative | NA |
| 13 | 60 | Male | 3 | AMS with seizure | 1 | 62.2* | Unilateral hippocampus | NA |
| 14 | 70 | Female | 3 | AMS with seizure | 9* | 114.4* | Bilateral temporal lobes and unilateral midbrain | NA |
| 15 | 61 | Female | 0 | AMS with seizure | 4 | 30.0 | Negative | Positive |
| 16 | 22 | Male | 0 | AMS with ataxia | 1 | 22.0 | Negative | Positive |
| 17 | 54 | Female | 3 | AMS with aphasia | 1 | 15.6 | Negative | Positive |
| 18 | 19 | Male | 9 | AMS with seizure and increased muscle tone | 1 | 16.0 | Negative | NA |
| 19 | 39 | Male | 22 | AMS with decreased hearing | 1 | 47.0* | Negative | Positive |
| 20 | 41 | Male | 4 | AMS with increased muscle tone | 14* | 53.6* | Unilateral pontine and deep white matter | Negative |
| 21 | 68 | Male | 0 | AMS | 23* | 541.0* | Negative | Positive |
| 22 | 68 | Male | 1 | AMS with seizure, dysarthria, and dysphagia | 2 | 37.0 | Negative | Negative |
| 23 | 25 | Male | <1w | AMS | 13* | 24.2 | Unilateral hippocampus | NA |
| 24 | 53 | Male | 0 | AMS with unilateral facial paralysis and ataxia | 58* | 29.4 | Negative | Positive |
| 25 | 51 | Male | 12 | AMS with dysarthria, hypaesthesia, and involuntary movement | 4 | 37.8 | Unilateral parietal lobe (CT) | NA |
| 26 | 38 | Female | 15 | AMS with seizure | 8* | 50.6* | Multiple regions | Negative |
| 27 | 32 | Female | 0 | AMS | 181* | 450.0* | Negative | Positive |
| 28 | 42 | Male | 0 | AMS with involuntary movement | 494* | 1328.0* | Negative | NA |
| 29 | 24 | Male | 0 | AMS with seizure, aphasia, ataxia, and involuntary movement | 1 | 33.5 | Negative | Negative |
| 30 | 66 | Female | 0 | AMS with aphasia and focal motor deficit | 8* | 17.4 | Negative | NA |
| 31 | 38 | Female | 0 | AMS with dysarthria | 1 | 17.9 | Bilateral basal ganglia | Negative |
| 32 | 51 | Female | 15 | AMS with focal motor deficit | 4 | 28.5 | Multiple regions | NA |
| 33 | 18 | Male | 3 | AMS with seizure and ataxia | 1 | 19.9 | Negative | NA |
| 34 | 41 | Male | 8 | AMS with seizure and involuntary movements | 1 | 37.1 | Negative | NA |
| 35 | 35 | Male | <1w | AMS with seizure, focal motor deficit, and ataxia | 2 | 19.6 | Negative | NA |
| 36 | 45 | Female | 34 | AMS with focal motor deficit and ataxia | 1 | 53.1* | Multiple regions | NA |

Onset refers to days before (negative values) or after (positive values) the onset of confirmed COVID-19 infection. Abbreviations: AMS, altered mental state; CSF, cerebrospinal fluid; NA, not available.
